# Codon Usage Domains over Bacterial Chromosomes

**DOI:** 10.1371/journal.pcbi.0020037

**Published:** 2006-04-21

**Authors:** Marc Bailly-Bechet, Antoine Danchin, Mudassar Iqbal, Matteo Marsili, Massimo Vergassola

**Affiliations:** 1 CNRS URA 2171, Institute Pasteur, Unité Génétique in silico, Paris, France; 2 CNRS URA 2171, Institute Pasteur, Unité Génétique des Génomes Bactériens, Paris, France; 3 Abdus Salam International Center Theoretical Physics, Trieste, Italy; 4 Computing Laboratory, University of Kent, Canterbury, Kent, United Kingdom; Max Planck Institute for Molecular Genetics, Germany

## Abstract

The geography of codon bias distributions over prokaryotic genomes and its impact upon chromosomal organization are analyzed. To this aim, we introduce a clustering method based on information theory, specifically designed to cluster genes according to their codon usage and apply it to the coding sequences of Escherichia coli and Bacillus subtilis. One of the clusters identified in each of the organisms is found to be related to expression levels, as expected, but other groups feature an over-representation of genes belonging to different functional groups, namely horizontally transferred genes, motility, and intermediary metabolism. Furthermore, we show that genes with a similar bias tend to be close to each other on the chromosome and organized in coherent domains, more extended than operons, demonstrating a role of translation in structuring bacterial chromosomes. It is argued that a sizeable contribution to this effect comes from the dynamical compartimentalization induced by the recycling of tRNAs, leading to gene expression rates dependent on their genomic and expression context.

## Synopsis

Genomic sequencing projects are clearly showing that cellular components are not randomly encoded over bacterial chromosomes. Order arises for a variety of reasons. Bailly-Bechet and colleagues focused here on the role of translation in shaping bacterial chromosomes. Due to degeneracy of the genetic code, each amino acid can be encoded by multiple codons. Gene encoding is not random, though, and, depending on the genes, some codons are preferred to their synonyms. This is the so-called codon bias phenomenon. The authors analyzed the usage of synonymous codons for protein encoding and its geography over bacterial chromosomes. They found that genes sharing similar codon bias tend to be close to each other on the chromosome, in coherent patches more extended than transcriptional units. Their hypothesis is that those correlations in codon bias enable the cell to locally recycle tRNAs employed during translation, reducing stalling of the ribosomes due to rare tRNAs. This also entails a dependence of expression rates of a gene on its chromosomal context. Furthermore, their analysis made clear that genes involved in anabolic pathways, mainly active when the cell is starving, have a similar codon usage, and that they are encoded on the lagging strand of DNA. They hypothesize that this is due to relative translation efficiency of the lagging strand as compared with the leading one, illustrating the role of translation in creating structural evolutionary constraints.

## Introduction

The degeneracy of the genetic code entails that all amino acids except methionine and tryptophan are encoded by multiple synonymous codons. The usage of synonymous codons is far from neutral, though, and strong biases in their frequencies were observed in the first genomic sequences (see [1]). A general relation of proportionality between bias and tRNA abundance was early remarked both in Escherichia coli and Saccharomyces cerevisiae for highly expressed genes [2–4]. For this class of genes, the bias is thought to be driven by the rapidity of the translation process and is quantified by a Codon Adaptation Index (CAI), gauged on the frequencies observed in ribosomal proteins and some additional genes, highly expressed under exponential growth conditions [5]. Highly and lowly expressed genes are clearly separated in two different groups by multivariate cluster analysis [6].

Expression levels do not exhaust the possible sources of selective pressures on protein encodings. For example, proteins synthesized under conditions of starvation for certain amino acids obey rather different principles of selection. Mazel and Marlière [7] showed that, under conditions of sulphur limitation, the most abundant proteins of the cyanobacterium *Calothrix* are encoded so as to reduce their sulphur requests. More recently, Elf et al. [8] have shown that when the codon reading is part of a control loop that regulates synthesis of a starved amino acid the codon choice seems to be as sensitive as possible to starvation.

Furthermore, a possible role of the translation kinetics and codon usage for a proper folding of the nascent protein was proposed by Thanaraj and Argos [9,10]. Finally, a whole class of genes known to have a specific type of bias is composed of horizontally transferred genes, as shown using multivariate correspondence analysis [11,12]. This remark was subsequently used to trace back the evolutive origin of outer membrane genes in E. coli [13] and to identify biases in the functions of horizontally transferred genes [14]. While general properties of codon usage have been considered in great detail, little information is available on the global organization of the bias over the chromosomes. This is the issue broached in the present paper. The methodology that we employ is to cluster genes according to their codon bias and analyze the resulting groups. This procedure has a twofold advantage.

First, it allows identifying groups of genes sharing a similar codon usage and, looking at their composition, inferring the possible causes of the observed biases. Second, information on the codon usage of the various genes is condensed into their cluster membership, whose correlations and distribution over the chromosome are most conveniently analyzed. General-purpose multivariate methods for clustering genes according to their codon usage have been reviewed by Perrière and Thioulouse [15], who raised a list of relevant points on their limitations. In particular, the counts of the various codons for the different genes are highly variable and might be rather low for some amino acids.

Standard choices for the distance between couples of genes are therefore doomed to strongly fluctuate and possibly to lead to artifacts. Furthermore, no objective criterion is usually provided to choose the number of clusters. Those points motivated us to devise a new clustering method, specific to the problem of codon bias analysis. The procedure is presented in detail in the Materials and Methods section. The basic idea is to assign *all* coding sequences of a genome to *S* clusters and look for the best partition in terms of information content. Each cluster is characterized by its own distribution of codon usage, i.e., the probabilities of using a given codon to encode a given amino acid, and the distribution is supposed to be common to all the coding sequences composing the cluster. The number of clusters *S* is determined by a systematic criterion based on cluster stability.

The Results section presents the application of the new method to the coding sequences of the two most-studied representatives of gram-negative and gram-positive bacteria, E. coli and Bacillus subtilis. The analysis of the clusters so identified indicate that they are, both statistically and biologically, highly consistent and that our clustering method significantly improves over previous works. The biological significance and implications of the clusters are further investigated in the Discussion section, where we discuss the possible mechanisms yielding the strong and extended correlations in codon bias observed over the chromosomes and the implications for chromosomal organization.

## Results

The clusters obtained by our new clustering method for E. coli and *B. subtilis,* and their geography over the chromosomes, will be presented in the following subsections.

### Cluster Structures in E. coli and B. subtilis

**Figure 1:**
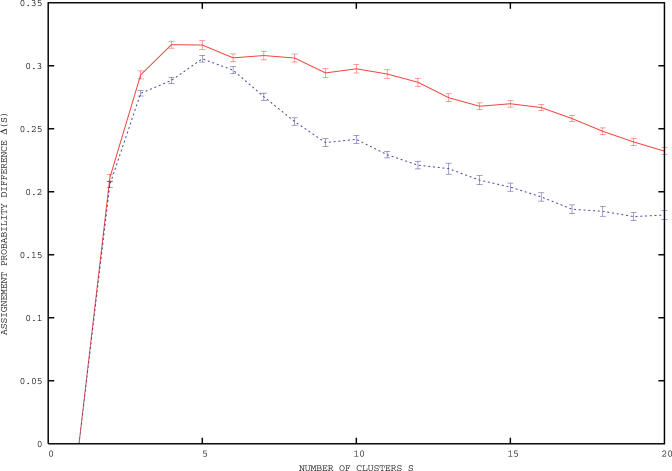
The Cluster Stability Curves, Quantified by the Difference Δ(*S*) = *B*(*S*) *− B_random_*(*S*) of the Assignment Probabilities Defined in the Body of the Text, versus the Number of Clusters *S* The curves are for B. subtilis (dashed blue) and E. coli K12 (solid red). The retained number of clusters corresponds to the maximum of the stability curve.

**Figure 2:**
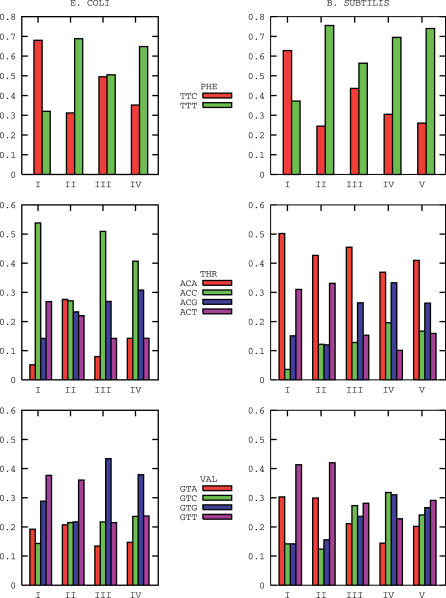
Average Posterior Probabilities of Usage for the Codons of Phenylalanine, Threonine, and Valine in the Clusters Identified for E. coli K12 and B. subtilis E. coli K12, left column; *B. subtilis,* right column. Clusters are identified by a roman number on the *x*-axis. The corresponding standard deviations are on the order of a few percent of the average values.

**Figure 3:**
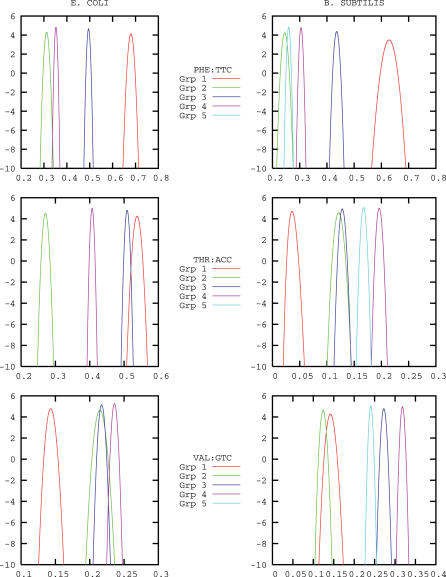
The Posterior Probability Distributions for Three Representative Codons: TTC (Phenylalanine), ACC (Threonine), and GTC (Valine) in the Clusters That We Identified for E. coli K12 and B. subtilis E. coli K12, left column; *B. subtilis,* right column. The curves are meant to show that the clusters are well separated by the combined information on the various codons.

The number of clusters identified for E. coli K12 and B. subtilis are four and five, respectively, as shown by the curves in Figure 1. In Figure 2, the posterior average probabilities of codon usage for phenylalanine, threonine, and valine are reported. These three amino acids are chosen as others are either more rare (C,H,Y), have their codons enriched in GC bases (A,G,P), are affected by deamination processes (N,Q), or have a biased distribution along the proteins (D,E,K) [16]. Probabilities of usage for all amino acids are reported in Tables S1 and S2. In Figure 3, we report the posterior probability distributions for three codons of the previously mentioned amino acids phenylalanine, threonine, and valine. The curves show that the clusters are indeed well-separated and that the separation arises by the combined effect of the various codons is not dominated by a single one. An important point is that the clustering is not due to trivial differences in GC content between genes, as the average GC content of the genes in the various clusters varies only from 49.28% to 49.32% in *E. coli,* and from 42.10% to 42.18% in B. subtilis.

Strong indications in favor of the biological significance of the clusters stem from three different statistics: the Codon Adaptation Index (CAI), the distribution of the cluster memberships among genes composing operons, and their distribution among genes coding for proteins intervening into a common metabolic pathway. As for the CAI [5], genes used to gauge the index are all highly expressed and share codon usages strongly biased toward the most abundant tRNA iso-acceptors expressed under exponential growth conditions [2,3]. Those genes are therefore expected to co-cluster. Indeed, we find that the great majority (32/59) of genes used to gauge the CAI index [17] for B. subtilis belongs to the first group in Figure 2 (the complete list of the cluster memberships for the CAI genes of B. subtilis is available in Table S5). The statistical significance of the event is very high (gathering 32 genes or more in the first cluster has a probability of 10^−29^ to occur by chance).

For E. coli K12, the co-clustering of its genes used to gauge the CAI index [17] is even stronger, as they all belong to the first group in Figure 2, and the event has a probability 10^−44^ to occur by chance. Genes belonging to operons are co-transcribed in a polycistronic mRNA molecule, and they are then expected to share similar pressures on the translation process. Exceptions and special cases ought to be expected for various reasons: genes transcribed from alternative promoters, different folding kinetics and expression levels, and differential regulation of the translation process among the various genes of the operon, etc. For example, genes within the *gal* operon of E. coli are involved in functions only partially overlapping and their polarity is regulated by the Spot42 noncoding RNA [18].

**Figure 4:**
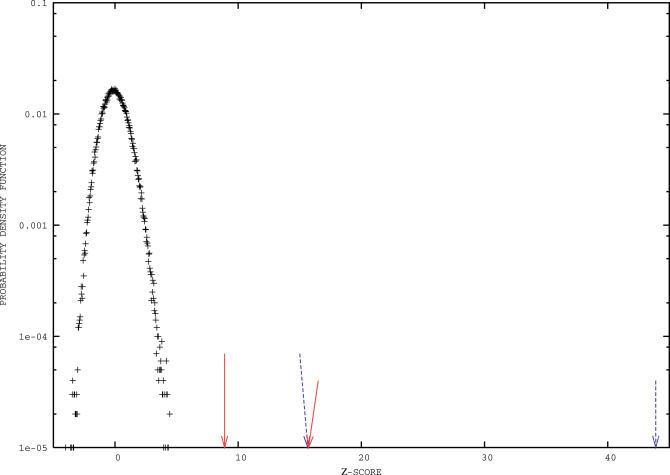
A Centered Gaussian Probability Distribution of Unit Variance (Black), Corresponding to the Random Distribution Obtained in the Null Models, and the Values Actually Observed in Our Clusters (Arrows) Values reported on the abscissae are *z*-scores, i.e., the deviations to the mean normalized by the standard deviation. Red solid and blue dashed arrows correspond to E. coli K12 and *B. subtilis,* respectively. Short arrows point to the values of the *z*-scores that we measure for the fraction of pairs of genes within a common operon and belonging to the same cluster. Long arrows refer to the same quantities for pairs of genes within a common metabolic pathway. Note that, as the Gaussian distribution is meant to show, our *z*-scores are highly significant, e.g., *z*_score_, ≥ 8 ↦ probability = 6 *×* 10^−16^ to occur by chance. See also that values of the *z*-scores previously obtained, using general-purpose clustering methods, were much smaller: 5.30 and 3.29, for operons and metabolic pathways, respectively.

It is, however, expected that at least on a global statistical level, genes within a common operon should display a tendency to share a similar usage of codons, i.e., co-cluster. The same tendency is expected for genes belonging to common metabolic pathways, as their expression tends to be correlated, namely in time. Indeed, considering the list of known operons and metabolic pathways and comparing their cluster memberships to null models generated as described in Materials and Methods, we obtain the results shown in Figure 4. Genes belonging to common operons and/or metabolic pathways have a strong tendency to share the same cluster membership. The observed values of the *z*-scores (8.9, 15.7 for *E. coli,* and 15.6, 43.9 for B. subtilis) correspond to extremely low *p*-values (3 *×* 10^−19^, 8 *×* 10^−56^, 4 *×* 10^−55^, and exp (*−*968.3), respectively), and our clustering method manifestly allows significant improvements over previous results obtained by general-purpose multivariate clustering methods [11,12].

### Functional Properties and Distribution over the Strands of Genes in the Clusters

Clusters identified in the previous subsection have marked properties regarding the functional categories of their genes. As previously shown, the first groups in Figure 2 contain an overwhelming number of highly expressed genes involved in translation, ribosomal structure, and biogenesis. This was largely expected on the basis of known results [2,3,5]. More interestingly, other clusters, too, have quite specific properties in terms of the functional categories of their composing genes. A systematic analysis is performed using COG functional annotations [19] and looking at the composition of the various clusters. Deviations from the behavior expected by chance are assessed using artificial chromosomes generated as described in the Materials and Methods section. The results are reported in Tables S3 and S4.

A first class of genes whose distribution is highly nonhomogeneous across groups is that of genes poorly characterized and/or of unknown function (COG classes −, R, and S). Indeed, a striking excess of those genes is found in the second groups of both B. subtilis and E. coli K12. A more detailed analysis reveals that a great deal of them are in prophage, mobile, and horizontally transferred regions. Furthermore, when the two previous groups are compared to the “horizontally transferred” groups previously found in [11,12], a large overlap is found. This confirms the special usage of codons by horizontally transferred genes and the possibility of detecting them by their codon bias.

Another class of genes which we find to be biased is composed of genes involved in the motility of the cell (COG class N). They also feature a peculiar usage of the codons, appearing preferentially in the fifth cluster of B. subtilis. A third, large class of genes with a special distribution among the clusters is composed of metabolic synthesis and transport genes. The third group in Figure 2 for B. subtilis features indeed a significant excess of genes belonging to the COG categories C (energy production and conversion), E (amino acid transport and metabolism), and F (nucleotide transport and metabolism).

The fourth group also contains an excess of genes involved in carbohydrate transport and metabolism (the COG G category). Metabolic genes in E. coli also tend to gather in the third group, with significant overabundances of genes belonging to the COG categories C, E, H (coenzyme transport and metabolism), and P (inorganic ion transport and metabolism). Deviations to the random values for those classes are highly significant, with *z*-scores all larger than 3.4 and soaring up to 6.5.

In addition to genes coding for cytoplasmic metabolic genes, we find that many genes in this class code for transport systems. The corresponding proteins are associated with the bacterial envelope, a compartment that is significantly smaller in volume than the cytoplasm, asking for a consistently smaller number of individual proteins. Whether this quantitative feature is relevant to our observation remains to be seen. The functional properties just presented appear even more relevant if coupled with the analysis of the strand of the genes composing the clusters, i.e., their direction of transcription as compared with the direction of the replication fork. The distribution of genes over the two strands is a major feature of prokaryotic genomes, with a dramatic asymmetry in *B. subtilis,* where about 74% of the genes are transcribed in the same direction as the replication forks, i.e., located on the leading strand of the chromosome. The global effect is weaker in E. coli (about 55% of the genes are on the leading strand), but specific classes of genes are known to be strongly biased, e.g., essential genes on the leading strand [20].

**Figure 5:**
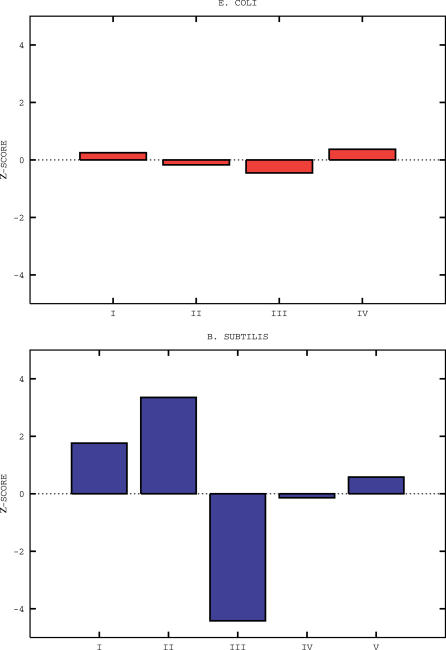
The Distribution of the Number of Genes on the Leading Strand for the Clusters of E. coli K12 and B. subtilis E. coli is shown on the top graph, and B. subtilis is shown on the lower graph. Clusters are identified by a roman number on the *x*-axis, and *z*-scores relative to null models are indicated on the *y*-axis. Note the depletion of leading strand genes in the third cluster of B. subtilis.

While most clusters do not feature any significant preference for a particular strand, a few of them do, as shown in Figure 5. The most relevant biologically (see the discussion in the next section) is the strong overabundance of genes on the lagging strand found in the third cluster of B. subtilis. The strand asymmetry emerges also from the codon usage posterior probabilities (see Table S2). Indeed, leading and lagging strands have a marked excess of guanines and cytosines, respectively, violating the naïve expectation of an equidistribution [21,22]. The reason is that the two DNA strands are exposed as single strands for quite different lags during replication, due to the kinetics of the formation and ligation of the Okazaki fragments. That induces different rates and dynamics in the mutation and repair processes, eventually leading to the observed G/C asymmetry (see [23,24] and references therein). In conclusion, the third cluster of B. subtilis is the same as previously shown to contain an excess of genes involved in energy production and transport and metabolism of nucleotides, carbohydrates, amino acids, and metabolites.

### Correlations in Codon Usage over the Chromosomes

**Figure 6:**
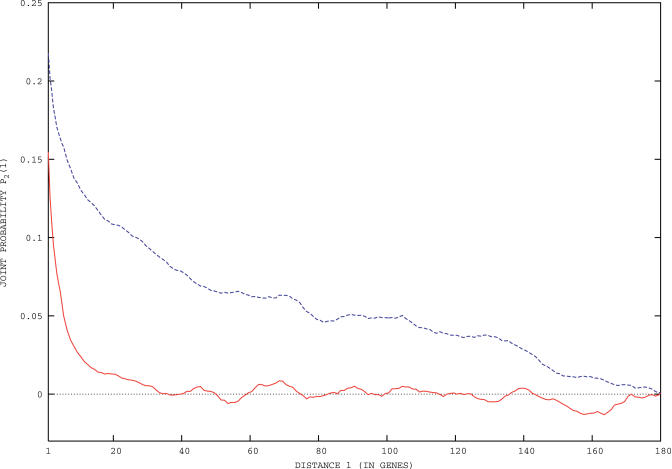
The Correlation Function (1) of Cluster Memberships versus the Distance among Genes for B. subtilis and *E.coli* K12 Blue dashed lines are for *B. subtilis,* and red solid lines are for *E. coli.*

**Figure 7:**
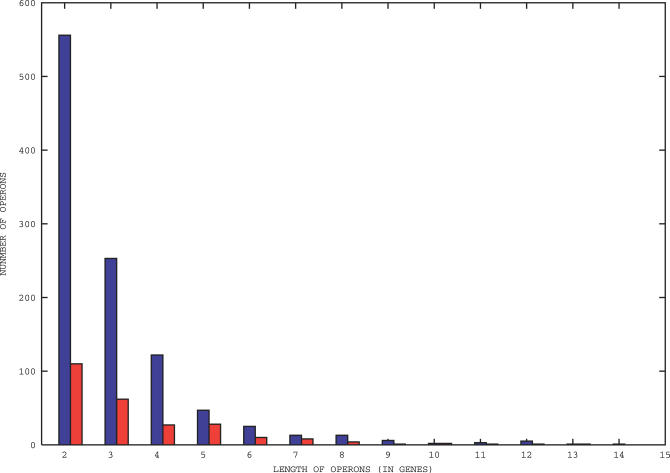
The Histograms of Lengths of the Known Operons for B. subtilis and E. coli K12 Blue boxes are for *B. subtilis,* and red boxes are for E. coli K12.

Let us now consider the spatial correlations of cluster memberships along the genomic sequence. The simplest relevant statistic to quantify them is the joint probability that two genes, *g* and *g* + *l,* belong to the same cluster (*s_g_* = *s_g+l_*):


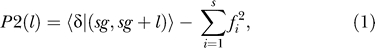


where δ is the Kronecker delta function, *S* is the number of clusters and *fi* is the total fraction of genes belonging to the *i*-th cluster. The asymptotic value 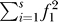 , corresponding to decorrelation between the two positions, is subtracted to ensure that the function in Equation 1 decays to zero at large distances, as shown in Figure 6. Note that genes are ranked in increasing order with respect to their translation start, so that *l* coincides with their spacing. In Figure 6 correlations are very extended, especially for *B. subtilis,* witnessing a similar usage of the code within rather wide domains. The most immediate possible explanation is that correlations might be simply due to constraints imposed by operons. This is, however, not the case, as shown in Figure 7. Lengths of the operons are way too short to account for the correlation lengths observed in Figure 6. Even in the case of *E. coli,* correlations extend to lengths five times larger than the average length of the operons. Alternative arguments leading to the same conclusion are presented in Figures S1 and S2. Another natural thought is that the extended correlations in Figure 6 might reflect the G/C skewed distribution. We have, however, previously remarked that variations in the GC content of the clusters are very tiny, ruling out this simple possibility. Even leaving statistics aside, a direct inspection reveals that cluster memberships are organized in coherent domains, often extending beyond the limits of known operons. Prophages and horizontally transferred regions contribute to the trend, but the coherence is not restricted to those cases and does not seem to be associated with any particular functional class or regions of the chromosome. A possible explanation of the phenomenon will be proposed in the Discussion section.

### Discussion

Two results obtained that were explained in the section above seem particularly relevant to the organization of bacterial chromosomes and will be discussed here in a more extended way. The first is the extent of codon bias correlations observed in Figure 6, much longer than what could be accounted for by operons. Theoretically, the existence of long-range correlations among individual nucleotides is well-known (see [25–28]). At a higher level of organization, sequence domains of order higher than operons, dubbed über-operons or super-operons, have been evidenced in the literature [29,30]. It has been noted by Rogozin et al. [30] that sizeable minorities in super-operons do not have any obvious functional relationship to the rest of the neighborhood, but seem to “car pool” with it.

Experimentally, recent data demonstrate that bacterial chromosomes have a definite spatial arrangement and are organized in macrodomains [31]. Macrodomains are playing a major role in the nucleoid organization and have strong practical implications for tentatively minimizing the size of artificial genomes [32]. A relation between these structural macrodomains and the sequence domains discussed here is plausible but remains to be demonstrated. Our results go in the direction of domains of order higher than operons. The novel point brought by our analysis is the explicit connection made between these structures and the translation process. Indeed, Figure 6 demonstrates that neighbouring genes tend to have a similar bias in their codon usage and suggests that the corresponding mRNAs reciprocally affect their translation processes. In other words, efficiency and rates of translation of mRNAs might not be a function of the mRNA only, but be quite sensitive to its genomic and expression context, too.

A sense of the relevance of these context effects might be drawn from a few simple estimates. Their goal is to assess the importance of tRNA recycling effects and the *rationale* is as follows: if the concentration of tRNAs turned out to be limiting, it would be sensible to propose that neighbouring genes tend to use codons similarly, so as to maximize their reciprocal recycling of tRNAs; conversely, if tRNAs turned out to be very abundant, it would be hard to imagine that such effects might be of any relevance. We shall suppose that tRNAs diffuse within the cell. No specific value for their diffusivity will be needed and, even though the hypothesis is likely to be an oversimplification, it should allow capturing the right orders of magnitude. The size of the cell is taken as *Scell* ≃ 1 μm and the number of ribosomes *N_ribo_* ≃ 20,000 − 60,000. The number of copies *n* of the various species of tRNAs in E. coli have been measured by Dong et al. [33] and vary from a few hundreds to several thousands. The typical distance between synonymous tRNAs is simply estimated as *lc* ≃ *S_cell_*/*n* ^1/3^.

Let us now consider a tRNA that has just been employed somewhere in the elongation of a polypeptide chain and estimate the distance it will travel before being caught again by another ribosome. This is a classical calculation of diffusion-limited cross section, already employed in the biophysical literature to estimate the time for a transcription factor to find its target over DNA (see, e.g., [34] for a recent review). The result that we shall need is Smoluchowski's probability, 1 − 4π*b/r,* that a particle at an initial distance *r* from a target of size *b* diffuses away from it without being caught. In our case, the targets are the ribosomes and their number will grow with *r* as *N_ribo_* (*r/S_cell_*)^3^. The recycling length *l_recy_,* i.e., the distance *r* such that it is practically certain that the tRNA will be caught again by a ribosome, is obtained from the relation *N_ribo_* (*r*/*S_cell_*)^3^ × 4π*b*/*r* ≃ *0*(1). Conservatively assuming the target size *b* to be 1/10 of the size of the ribosomes (≃25 nm), we come up with an estimate of *l_recy_* ≃ *0*(0.1μm), comparable to the typical distance *l_c_* for tRNAs having a thousand copies in the cell. The upshot is that the recycling of tRNAs is of importance for many of them, namely those rare and moderately abundant.

Notwithstanding the crudeness of previous estimates, there are biological indications supporting the conclusion that rare tRNAs might indeed be limiting in the translation process. Early experiments by Varenne and co-workers showed significant pauses at codons associated with rare tRNAs [35]. Another suggestive indication is the high concentration of tmRNAs, the surrogate tRNAs that append a peptide tag to nascent polypeptides and “rescue” stalled ribosomes, promoting rapid degradation of tagged proteins. Their number of copies in the cell is abundant, on the order of 13,000 [36], and it was recently shown that those concentrations are safely well above saturation [37]. This witnesses the importance of ribosome stalling events, e.g., due to delays in the recruitment of rare tRNAs. The concentration of tmRNAs in the cell is in fact strikingly higher as compared with that of rare tRNAs. This suggests that some recycling of rare tRNAs ought to be at work to create higher transient local aggregations of tRNAs, compensating for their much lower average value over the whole cell. The experimental observations reported in [38], of channeling and slowing-down of the diffusion of macromolecular components of the translation apparatus, might be relevant in that respect.

The combination of all previous arguments leads us to propose a role for the codon bias domains over bacterial chromosomes that we have found, viz., that they allow a coordinated control of the expression levels of nearby genes and increase their reciprocal tRNA recyclings, so as to alleviate stalling effects. A very interesting experiment to test these ideas, yet quite difficult to design, would consist in reliably measuring possible dependencies of mRNA translation rates on their genomic and expression context. The second intriguing result presented here is the fact that anabolic genes in B. subtilis tend to aggregate in a single cluster and that this cluster features an excess of genes over the lagging strand. Specifically, genes in the aforementioned cluster belong to the functional classes of transport and metabolism of amino acids, carbohydrates, and nucleotides.

We shall argue that these observations are in fact strongly related and driven by the following biological mechanisms. First, genes of the previous functional classes are likely to be mostly expressed and employed in poor media, where the bacterium cannot easily import its essential metabolites from the external medium and is obliged to finely scavenge its environment and/or to synthesize them. These processes of synthesis will induce a long lag between two successive replications, in sharp contrast to the case of a rich medium. There, generation times are so rapid that bacteria are essentially always replicating, and several replicative forks are progressing at the same time over the chromosome.

Second, head-on collisions between transcriptional and replicative machineries are known to be deleterious to the proper functioning of the cell. The dynamics of the interaction between DNA and RNA polymerases have been thoroughly investigated [39–41]. Replication elongation is found to be weakly affected by co-directional transcription, whilst head-on collisions induce a severe inhibition of the replicative fork progression. It is therefore quite sensible that a strong selective pressure is at work in prokaryotic genomes to reduce deleterious effects of head-on collisions. Those are the major cause for the strand asymmetry observed in prokaryotic genomes and, in particular, of essential genes [20]. Pressure to avoid head-on collisions seems particularly cogent in *B. subtilis,* where about 74% of all genes are found on the leading strand.

Combining the two previous remarks provides a clue to the observed preferential positioning of anabolic genes on the lagging strand: due to longer replication times in poor media than in rich media, genes expressed in the former will be subject to a relatively lower pressure to be on the leading strand as compared with genes active in rich media. Furthermore, transport proteins are located in the membranes or the periplasm, compartments that are significantly smaller than the cytoplasm, asking therefore for a significantly lower number of individual proteins of that type. There is even a strong selection pressure against too high expression of membrane proteins as reflected by the toxicity of overexpression of the corresponding genes (see [42] for a review of significant data in the domain). The resulting differential selective pressures might then contribute to the observed strand asymmetry.

This hypothesis can be directly tested by measuring the expression levels of genes, e.g., in a transcriptome experiment. The only caveat and precaution to be taken is that bacteria in the cultures should be synchronized with respect to their cell cycle, and the expression levels not be averaged out over the cell cycle, as in standard in experiments. Averaging is clearly inappropriate for genes whose expression levels strongly depend on the cycle of the cell, e.g., for the classical example of *ftsZ* [43]. Tracking the expression of those genes requires working with synchronous cultures and specific methods to meet this goal (see [44] for a review). Novel possibilities recently advanced [45] appear particularly promising and appropriate for an experimental test of the hypothesis suggested by our results, namely that genes encoded in the lagging strand direction are preferentially expressed in inter-replicative phases.

## Materials and Methods

Given a set of *G* genes, all supposed to be translated according to the standard genetic code, our aim is to find their best partition into *S* clusters. More precisely, each cluster is supposed to have a common distribution of codon usage, i.e., nucleotide sequences of genes belonging to the same cluster are all supposed to be encoded with that common distribution. Our goal is to determine the cluster partition that best describes the observed counts of codons. Note that the number *S* of clusters is unknown and ought to be found, too. As shown in the following subsections, we shall weight the various cluster configurations by the information that they encode on the codon usage probability distributions. We shall first derive the expression of the cluster information content in terms of the codon counts. Next, we shall describe how the configurations yielding the maximum information are sought numerically and how the method here compares with methods previously employed in the literature. Finally, we shall analyze the stability of the clusters so identified and provide a quantitative criterion for choosing the number of clusters. The last subsection is a brief description of the procedures to generate random artificial chromosomes as null models.

### Gathering information on codon usage distributions.

The distribution of codon usage for the *s*-th cluster *C_s_* is parameterized by the set of probabilities 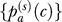 that codon *c* be used to encode amino acid *a*. The degree of degeneracy for the *a*-th amino acid is denoted by *qa,* e.g., the index *c* runs from 1 to *q_a_* = 4 for glycine and *q_a_* = 2 for phenylalanine. The amino acids to be clustered are those admitting multiple encodings, so that methionine and tryptophan can be excluded without any loss of generality. The index *a* then runs up to *A* = 18. A priori, the only information available is that amino acids might be encoded by any one of their synonymous codons. This state of ignorance is best described by a uniform prior distribution:


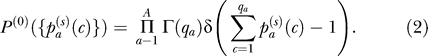


Dirac's *δ* function in Equation 2 imposes the constraint that, for each amino acid, the sum of the probabilities over synonymous codons is normalized to unity. Euler's Γ function ensures the normalization of the probability distribution, as can be easily checked using the general formula (see, e.g., [46]):


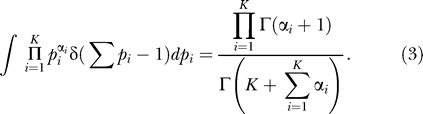


The uniform prior Equation 2 appears more appropriate to our situation than a prior uniform in the logarithms of the probabilities (see, e.g., [47]) as we know from the genetic code that synonymous codons are a priori all possible. Choosing a log-uniform prior would not, at any rate, modify substantially the results presented in the sequel. A posteriori, observing the codon counts of the genes assigned to the *s*-th cluster *C_s_,* we can infer its posterior distribution of codon usage as:


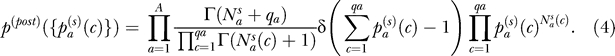


Here, 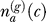 is the number of times codons of type *c* are used to code for amino acid *a* in gene *g ∈ C_s_* and we have used the shortcut notations: 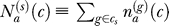 for the total number of times codons of type *c* are used for amino acids of type *a* in the *s*-th cluster and 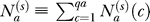 for the total number of occurrence of amino acid *a* in cluster *s*. Equation 4 is an instance of Bayes theorem: the prior is given by Equation 2 and the likelihood that codon counts of gene *g* be generated with the probability distribution of cluster *s* is a product of multinomials of order *q_a_*:


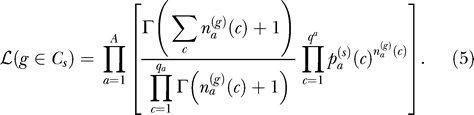


Information acquired on the codon usage distributions of the clusters is defined in terms of the classical Kullback-Leibler relative entropy between the posterior and the prior distribution (see, e.g., [48]) as:


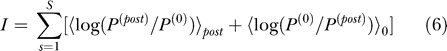


where the symbols *P*_0_ and *P*_post_ denote the averages with respect to the prior and the posterior distributions Equations 2 and 4, respectively. The information in Equation 6 can be calculated analytically and expressed in a simple form as a function of codon counts. To that purpose, it is sufficient to use the identity: 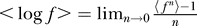 and Equation 3 to compute the resulting averages. The final expression is:


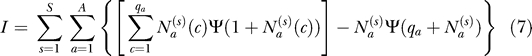


where we have omitted for simplicity constant terms, i.e., those which do not depend on the cluster configurations. The logarithmic derivatives Ψ of the Euler Γ function are calculated using the well-known formula [49]: 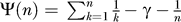 , with *γ* = 0.5772 … being Euler's constant. For each number of clusters *S,* we aim at identifying that assignment of the *G* genes to the *S* clusters that maximizes the information in Equation 7. It is worth noting that optimizing an entropy function is quite natural for our problem. Indeed, for G ≫ 1 and clusters sufficiently populated, posterior probability distributions are inferred from very long sequences of symbols, whose alphabet is defined by the set of synonymous codons. Since the empirical frequencies of codon usage are the types of the resulting sequences and their fluctuations are controlled by large deviation asymptotics (see chapter 12 in [48]), the entropy of the underlying probability distributions appears indeed as an appropriate quantity to consider.

### Numerical implementation and comparison to other methods.

We tried several methods to optimize the information in Equation 7, and the upshot is that its landscape in biological applications considered here is not particularly rough. This permits using a simple and rapid iterative method, based on a combination of hierarchical clustering and *k*-means [50,51]. The hierarchical clustering algorithm starts from clusters composed of individual genes and iteratively proceeds upward to generate optimal configurations for each possible *S* number of clusters. Iterations are based on the two following steps: 1) pairs of clusters are merged so as to get the maximal *I* in Equation 7; 2) the resulting configuration is taken as the initial condition for a *k*-means iteration (with *k* = *S* − 1). Elementary moves consist of changes in the cluster of assignment for each pair of genes. Moves increasing the score in Equation 7 are accepted and the procedure is repeated until the composition of the clusters does not change anymore. We have explicitly verified that other optimization methods, e.g., simulated annealing, are more time-consuming and do not modify the results in any substantial way. Let us conclude this subsection with a brief discussion on the choice and the comparison of our clustering method with previous works. As we have just discussed, the numerical method of optimization relies on the combination of two standard and commonly employed methods (*k*-means and hierarchical clustering). Conversely, the choice of the quantity to be optimized in Equation 7 is less usual. A more standard procedure would be to define a distance among pairs of genes and then minimize the sum of the intracluster distances. If counts of events are involved, as in codon bias clustering, classical choices for the pair-wise distance are the Euclidean distance between synonymous codon usage values or between percentage codon usage values [6] and the *χ^2^* metrics employed in [11,12]. Our motivation for going through the derivation leading to Equation 7 is that the counts of the codons feature a large variability over the various genes and that they can be rather low for some of the amino acids. The former implies that statistics such as percentage usages do capture average effects but are not quite rigorous in their accounting for the fluctuations: the same difference in percentage usage between two genes might indeed be highly significant or not, depending on the total number of counts involved. As for the *Â*_2_ metrics, its general relevance relies on the limit of a large number of counts, a hypothesis which is not verified for all amino acids in some of the genes. Possible consequences of enforcing *χ^2^* metrics with a low number of counts are described in [15], showing that the presence/absence of rare amino acids might dominate the clustering. Those problems might be fixed of course by restrictions on the length of the proteins, discarding rare amino acids, and, generally speaking, expert pre-and post-processing. This labor is reduced by maximizing Equation 7 and having a systematic criterion for the choice of the number of clusters (see the next section), even though the price to pay is a lengthier derivation. That was our reasoning in the choice of the clustering method and our motivation for favoring Equation 7 and the criterion presented in the next section for the number of clusters.

### Choosing the number of clusters.

The problem of how many clusters provide an appropriate description of the data is a classical issue in clustering [52,53]. A general perspective is given in [54] where the problem is reformulated in terms of an energy-versus-entropy competition. That elegantly demonstrates that the choice of the number of clusters is bound to depend on our level of description, condensed in [54] in the temperature of the system. The same fact is concretely indicated by Monte Carlo simulations by van Nimwegen et al. [55] for the clustering of transcription factor binding sites to predict regulons. When the space of possible configurations is sampled by Monte Carlo dynamics, clusters typically evaporate, drift, and fuse, and none of them lives forever, which makes a precise cluster membership identification quite problematic. A large variety of criteria for the choice of the number of clusters have been put forward in different problems [55–63]. In our case, since we shall be looking at functional categories of the genes composing the clusters, it is important to have a very reliable assignment of genes to clusters. We are therefore interested in imposing a criterion on the quality of the assignment and the stability of the clusters under reassignment. To this purpose, we shall employ a heuristic self-consistency criterion which has the advantage of being simple and rid of free parameters. A measure of the self-consistency in assigning gene *g* to cluster *s* is provided by the quantity: 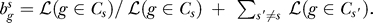 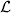 is the likelihood, defined in Equation 5, that the codon counts of gene *g* be generated with the probability distribution of cluster *s*. A value of 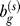 close to unity implies that the gene matches uniquely well the usage of cluster *s,* and we can then be confident that its assignment is meaningful.

Let us then consider a configuration of *S* clusters, identified as described in previous sections. The quality of the corresponding assignments is quantified by the geometric average 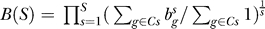 . Taking the arithmetic mean inside each cluster ensures that this measure is not dominated by individual genes, while the geometric mean across clusters ensures that none of them has poor assignments if *B*(*S*) is sufficiently close to unity. Rather than fixing an ad hoc threshold on *B*(*S*), we have found it more effective to compare the stability of clusters obtained for real data to those in null models. Specifically, we calculate the posterior probability distribution of the real dataset for a unique cluster, comprising all genes. This single-cluster probability distribution is then used to generate an artificial dataset: each gene has the same length and number of amino acids as in the real genome, but amino acids are randomly encoded with the previous single-cluster distribution. This procedure guarantees that the overall statistics of codon usage is preserved, yet no cluster structure is by definition present in artificial data. Artificial data are then clustered as previously described and the average *Brandom*(*S*) for these random data is computed over a sufficient number of realizations. The number of clusters retained is the one corresponding to the maximal difference Δ(*S*) = *B*(*S*) *− Brandom*(*S*), as shown in Figure 1 for B. subtilis and E. coli. Note that the assignment probabilities *B*(*S*) for the number of clusters corresponding to the maxima in Figure 1 are 0.9 and 0.94, witnessing a strong consistency and statistical significance of the clusters identified. We experimented on various datasets generated with a prescribed distribution of codon usage and found that the method just described efficiently recovers the correct structure of the clusters and their distributions of codon usage.

### Artificial chromosomes and null models.

Given *G* genes and the numbers *G_s_* (*s* = 1, … , *S*) of genes in the *S* clusters, random chromosomes were generated as follows. Initially, one has *G*_1_ cluster labels of the first type, *G*2 of the second type and so on (∑*s Gs* = *G*), and a label is picked randomly and attached to any one of the *G* genes. One then iterates the procedure, randomly attaching the remaining labels to yet unlabeled genes. This ensures that all finite-size effects and the size of the clusters are correctly taken into account. Null statistics were obtained measuring the quantity of interest, e.g., COG distributions, over artificial chromosomes and accumulating statistics over an ensemble of 100,000 realizations. The resulting distributions are close to Gaussian by the central limit theorem. It was therefore appropriate to weight the significance of the deviations between real data and random cases by the corresponding *z*-scores, i.e., the deviation of the observed value to the mean of the random case, normalized by its standard deviation.

### Data sources.

We downloaded the complete annotated genomes from the NCBI microbial genome database (ftp://ftp.ncbi.nih.gov/genomes/Bacteria). The list of genes used to gauge the Codon Adapatation Index (CAI) is downloaded from the EMGLib [17] Web site (http://pbil.univ-lyon1.fr/emglib/codon.html). The list of characterized transcripts for E. coli and B. subtilis is from [64, 65], while their metabolic pathways were taken from the KEGG: Kyoto Encyclopedia of Genes and Genomes database (http://www.genome.jp/kegg). The list of COG functional categories is discussed in [19] and is available at http://www.ncbi.nlm.nih.gov/COG.

## Supporting Information

Figure S1 Figure Correlation for Randomized Chromosome E. coli
Solid line is the correlation function of cluster memberships as in Figure 6, for E. coli. Dashed line is the correlation function obtained for a randomized genome where the intra-operon contributions to P2(l) are retained but those stemming from different operons are depleted. Specifically, the randomization procedure is realised as follows. Labels are randomly permuted within the operons, yet keeping the fractions of genes fixed. For example, an operon with three genes belonging to the cluster α and two to β is randomized into one with three genes belonging to cluster γ and two to δ, with γ and δ randomly chosen. The genes composing the operon will then give the same contribution to P_2_(l). However, since random permutations are independent among different operons, the inter-operon correlations will be depleted.
(25 KB EPS)

Figure S2 Figure Correlation for Randomized Chromosome B. subtilis
The same curves as in Figure S1, for *B. subtilis.* Note that the correlation length is strongly reduced in randomized genomes, witnessing the fact that constraints imposed by operons are not sufficient to account for the extended correlations observed in Figure 6.
(26 KB EPS)

Table S1 The Average Posterior Probabilities of Usage of the Synonymous Codons for the Four Clusters Identified in E. coli K12 (3 KB TEX)

Table S2 The Average Posterior Probabilities of Usage of the Synonymous Codons for the Five Clusters Identified in B. subtilis
(3 KB TEX)

Table S3 The Distribution of Genes among the Functional COG Classes for the Clusters Identified in E. coli
For each of the COG categories, the first line is the measured number of genes for that COG category, while the second line is the corresponding *z*-score (deviation to the number expected by chance, normalized by the standard deviation).
(2 KB TEX)

Table S4 The Distribution of Genes among the Functional COG Classes for the Clusters Identified in B. subtilis
For each of the COG categories, the first line is the measured number of genes for that COG category, while the second line is the corresponding *z-*score (deviation to the number expected by chance, normalized by the standard deviation).
(3 KB TEX)

Table S5 Repartition of the Genes Employed to Gauge the Codon Adaptation among the Clusters Identified in B. subtilis
Note the highly significant concentration in the first cluster. Genes used to gauge the CAI index for E. coli are all concentrated in the first cluster.
(28 KB PDF)

## Abbreviations

CAI: Codon Adaptation Index
