# Supplementary material for: Codon Usage Domains over Bacterial Chromosomes
Source: PLoS Comput Biol. 2006 Apr 21;2(4):e37. doi: 10.1371/journal.pcbi.0020037 (PMC1447655; doi:10.1371/journal.pcbi.0020037)
Supplement: Table S5 — Note the highly significant concentration in the first cluster. Genes used to gauge the CAI index for E. coli are all concentrated in the first cluster. (28 KB PDF) [file pcbi.0020037.st005.pdf]

Gene	Group	Gene	Group	Gene	Group	Gene	Group
abrB	1	rpsE	1	yflH	3	pdhC	1
rplJ	1	rpmD	1	yfhD	3	ylaI	4
rplL	1	rplO	1	cspB	1	ylaJ	5
rplC	1	rplM	1	yhcN	2	divIVA	3
rplD	1	rpsI	1	prsA	1	rpmB	1
rplB	1	ybfQ	3	yhfD	1	fliJ	2
rplP	1	ycdA	3	cotW	3	cotE	5
rpsH	1	ycnE	1	ykwD	1	ynzH	2
rplF	1	ydbN	4	yknT	5	ynzC	3
rplR	1	ydcN	2	ykzG	2	yonK	2
ypzA	4	eno	1	rpsT	1	cotG	1
yokF	2	yttA	3	cspD	1	ytlB	4
ypjD	5	yvcE	1	fer	1	yvzB	1
ysnF	5	ywhB	3	rpmI	1	yxeE	3
yscB	5	ahpC	1	rpsD	1		

Table 3: Repartition of the genes employed to gauge the Codon Adaptation Index [5], as in [17], among the clusters identified in *B. subtilis*. Note the highly significant concentration in the first cluster. Genes used to gauge the CAI index for *E. coli* are all concentrated in the first cluster.
